# Does the Scientific Literature on Fractures Support Evidence‐Based Physiotherapy? Mapping the Evidence on the PEDro Platform

**DOI:** 10.1002/pri.70110

**Published:** 2025-09-22

**Authors:** Aline Miranda Ferreira, Raquel Metzker Mendes Sugano, Ana Carolina Carmona Vendramim, Pedro Henrique Alberani Soares, Marisa de Cássia Registro Fonseca

**Affiliations:** ^1^ Clinics Hospital of Ribeirão Preto Medical School University of São Paulo Ribeirão Preto São Paulo Brazil; ^2^ Post‐Graduate Program in Orthopaedic Physiotherapy Clinics Hospital of Ribeirão Preto Medical School University of São Paulo Ribeirão Preto São Paulo Brazil; ^3^ Department of Health Sciences, Rehabilitation and Performance Program Ribeirão Preto Medical School University of São Paulo Ribeirão Preto São Paulo Brazil

**Keywords:** evidence‐based practice, fractures, musculoskeletal, orthopedic trauma, physiotherapy

## Abstract

**Background:**

Bone fractures impose significant healthcare costs and are among the musculoskeletal conditions most requiring rehabilitation. Evidence‐based practice (EBP) is the recommended approach for ensuring quality and consistency of care. However, does the scientific literature on fractures adequately support evidence‐based physiotherapy?

**Purpose:**

To map the profile of fracture studies indexed on the PEDro platform, focusing on study designs, body segments studied, publication decade, and geographical distribution.

**Methods:**

Two authors independently extracted data from the PEDro platform using the term fracture in the musculoskeletal and orthopedic subdiscipline filter. Studies without abstracts or full articles available and those not primarily focused on fractures were excluded.

**Results:**

Six hundred sixty‐seven met the inclusion criteria, representing 3.2% of all musculoskeletal and orthopedic studies. Twelve clinical practice guidelines, 141 systematic reviews and 514 CTs were identified. The studies prioritize the hip (35.7%) and wrist (23.1%). Clinical trials were concentrated in Europe (47.8%), Asia (24.9%), North America (21%), and Oceania (11%), with few studies from South America (1.2%), Africa (0.2%) and none from Central America. The average PEDro scale score was 5.35 ± 1.81, with a peak in study production observed in the 2010s.

**Conclusion:**

Fracture studies on the PEDro platform are limited in number and quality. Many segments lack clinical guidelines and systematic reviews or trials. The geographic distribution reflects disparities in scientific production, favoring developed regions. These findings underscore the need for improved research and dissemination efforts, particularly in regions with lower representation.

## 1 Introduction

Bone fractures are a major global health problem, exerting wide‐ranging clinical and economic impacts on individuals and healthcare systems. They are among the leading causes of years lived with disability, ranking second only to low back pain in some countries (https://vizhub.healthdata.org/rehabilitation/). Falls, road traffic injuries, and mechanical forces are the most frequent causes, followed by unintentional injuries and conflicts (GBD 2019 Fracture Collaborators et al. 2021). Beyond the immediate trauma, fractures result in functional limitations, chronic pain, and reduced physical capacity, leading to long‐term declines in quality of life (Cieza et al. 2021; Filip et al. 2022). They also impose significant economic costs through workplace absences, reduced productivity, and elevated treatment expenses (GBD 2019 Diseases and Injuries Collaborators 2020; Polinder et al. 2016). For these reasons, research focused on prevention and treatment is essential to reduce their burden and improve population health and well‐being.

The Rehabilitation 2030 initiative by the World Health Organization (WHO) includes “Building research capacity and expanding the availability of robust evidence for rehabilitation” among the 10 priority areas for action. Additionally, WHO included fractures in the musculoskeletal Package of Interventions for Rehabilitation (2023), for which a workforce is needed (www.who.int).

Evidence‐based practice (EBP) and patient‐centered care ensure the quality and consistency of physiotherapy treatment (Scurlock‐Evans et al. 2014; Rauch et al. 2008).

The Physiotherapy Evidence Database (PEDro) is a free, internationally recognized resource that supports evidence‐based physiotherapy. It offers quick access to randomized controlled trials, systematic reviews, and clinical practice guidelines focused on physiotherapy interventions. Clinical trials at PEDro are quality rated, helping clinicians identify the best evidence for treatment. PEDro also had a slightly higher free full‐text access than PubMed, except for more recently published articles, and did not vary by geographic location. The database is widely used across many countries and regions with particularly high engagement in some areas, such as Brazil (Moseley et al. 2020, 2022).

While physiotherapy is crucial in fracture rehabilitation, it remains unclear whether the current scientific literature on fractures adequately supports evidence‐based practice. To address this gap, this study aims to analyze the profile of fracture‐related studies indexed on the PEDro platform, examining their design, body segment focus, publication trends, quality of trials, and geographical distribution.

## 2 Materials and Methods

### 2.1 Methods

A search was conducted on April 28, 2025, in the Physiotherapy Evidence Database (PEDro) using the term “fracture” combined with the musculoskeletal and orthopedic subdiscipline filters. These filters were selected to identify studies specifically related to bone fractures. All three study design categories available in PEDro: clinical trials, systematic reviews, and clinical practice guidelines were included. The search results were exported to Rayyan software where duplicate records were removed. No normalization procedures were applied, as the goal of the study was to describe the fracture‐related literature available in PEDro as presented by the platform's indexing system.

### 2.2 Selection Process

Two authors independently screened the titles and abstracts of all retrieved records to determine eligibility. Disagreements were resolved through open discussion and a consensus‐based approach. When the title and abstract did not provide sufficient information to determine eligibility, the full text was obtained and reviewed. Articles were excluded if the full text could not be located after consulting the database link and additional sources, or if fractures were not the central focus of the study.

### 2.3 Data Extraction and Analysis

Data from the included studies were extracted into a Microsoft Excel spreadsheet using predefined labels. Extracted variables included the number of studies per body segment, study design type, and publication decade. For clinical trials, PEDro scale scores and the geographical distribution of studies were also recorded. A stratification was performed to analyze the distribution of studies according to body segment, with the specific inclusion criteria for each segment presented in Table 1.

**TABLE 1:** Inclusion criteria for articles and categories by segment.

General fractures	Therapeutic resources; No predefined classifications (e.g. upper or lower limbs); multiple segment fractures
Spine	Spinal fractures; osteoporosis exclusively affecting the spine.
Thoracic	Rib or sternum fractures.
Facial	Facial fractures.
Shoulder	Clavicle; scapula; proximal humerus; fragility fractures specific to this segment.
Humerus shaft	Humerus shaft fractures.
Elbow	Distal humerus; proximal radius and ulna.
Ulna/radius shaft	Radius, ulna, or both shaft fractures.
Wrist and fingers	Distal fractures of the radius or ulna; carpal bones, metacarpals, and interphalangeal bones; fragility fractures in this area.
Pelvis	Pelvis including acetabulum.
Hip	Proximal femur; fragility fractures in this segment.
Femur shaft	Femur shaft fractures.
Knee	Distal femur fractures; patella; proximal tibia.
Tibia shafts	Tibia shaft fractures.
Ankle and foot	Distal fractures of the tibia or fibula; tarsal bones; metatarsals; toes.
Fragility	Fragility fractures involving more than one segment.

## 3 Results

The initial search retrieved 17,542 records from the musculoskeletal subdiscipline and 3432 records from the orthopedic subdiscipline in the PEDro database. Applying the term “fracture” yielded 666 records within the orthopedic filter and 163 within the musculoskeletal filter, totaling 829 records. After removing 89 duplicates in Rayyan, 740 records remained for screening. Of these, 71 were excluded because fractures were not the central theme, one was excluded due to the unavailability of the full text, and one was excluded because the article had been retracted. A total of 667 studies met the eligibility criteria and were included in the review, representing approximately 3.2% of all studies indexed in PEDro within the musculoskeletal and orthopedic subdisciplines.

A total of 12 clinical practice guidelines (CPGs), 141 systematic reviews, and 514 CTs were identified (Table 2). 343 (51.4%) articles were dedicated exclusively to the lower limb, while 211 (31.6%) were related to the upper limb. The remaining 113 (16,9%) articles dealt with spinal, fragility‐related, general, facial, and thoracic fractures.

**TABLE 2:** Distribution of studies by segment, study design, and mean and standard deviation of clinical trial scores.

Segment	N° articles	CPS	SR	CT's	Scores (CT's)
Hip	238	7	56	175	5.66 ± 1.56
Wrist and fingers	154	1	19	134	5.14 ± 1.90
Ankle and foot	65	0	10	55	4.85 ± 2.11
General fractures	42	0	22	20	4.35 ± 1.69
Shoulder	41	0	8	33	5.88 ± 1.96
Spine	35	0	8	27	5.50 ± 1.61
Fragility	32	4	12	16	5.88 ± 1.54
Tibial shaft	21	0	1	20	5.05 ± 2.15
Elbow	11	0	2	9	5.38 ± 1.19
Knee	8	0	1	7	5.43 ± 1.27
Femoral shaft	6	0	0	6	5.17 ± 1.47
Radius/Ulna shaft	5	0	0	5	4.0 ± 2.35
Pelvis	4	0	2	2	5 ± 2.83
Thoracic	2	0	0	2	7 ± 0
Facial	2	0	0	2	4 ± 1.41
Humeral shaft	1	0	0	1	5.35 ± 1.81
Total	667	12	141	514	5.35 ± 1.81

Abbreviations: CPS: Clinical Practice Guidelines, CTs: Clinical Trial, SR: Systematic Review.

### 3.1 Upper Limb Fractures

Wrist and finger fractures were the most studied upper limb condition, addressed in 154 articles (73% of upper limb studies), including 9.7% in elderly populations and 9.1% in children. Shoulder fractures were the focus of 41 studies (9.8% in elderly). Elbow fractures were reported in 11 studies, with 27.3% involving pediatric populations. Ulna or radius shaft fractures were examined in 5 studies, of which 60% involved children. The single study on humerus shaft fractures did not focus on a specific age group.

### 3.2 Lower Limb Fractures

Among the 343 studies on lower limb fractures, the hip was the most frequently investigated segment, accounting for 238 articles (69.4%), of which 68.5% focused on elderly populations. Ankle and foot fractures were addressed in 65 studies, with 3.1% involving children. Tibial shaft fractures were examined in 21 studies, 4.8% of which involved children. Knee fractures were reported in 8 studies, with 12.5% involving elderly populations. Femoral shaft fractures were the subject of 6 studies, all of which involved children. Pelvic fractures were investigated in 4 studies.

### 3.3 Spine, Elderly, and General Fractures

Other topics comprised 113 studies, including general fractures (42; 37.2%), spinal fractures (35; 31.0%), fragility‐related fractures (32; 28.3%), facial fractures (2; 1.8%) and thoracic fractures (2; 1.8%). Among the fragility‐related fracture studies, 43.8% focused on elderly populations. In spinal fracture studies, 20% involved elderly populations, while general fracture studies included 4.8% of elderly and 2.4% of pediatric populations. Studies on facial and thoracic fractures did not focus on a specific age group.

### 3.4 Decade of Publication

The number of studies increased progressively over time. Only 37 studies were published before 1990, followed by 62 in 1991–2000 and 192 in 2001–2010. The peak occurred in the decade from 2011 to 2020, with 258 studies (representing 36.8% of all included studies), reflecting growing interest in fracture‐related research during that period. Although the number dropped to 118 in the 2021–present interval, this decline may be attributed to the shorter elapsed time since publication.

**FIGURE 1:**
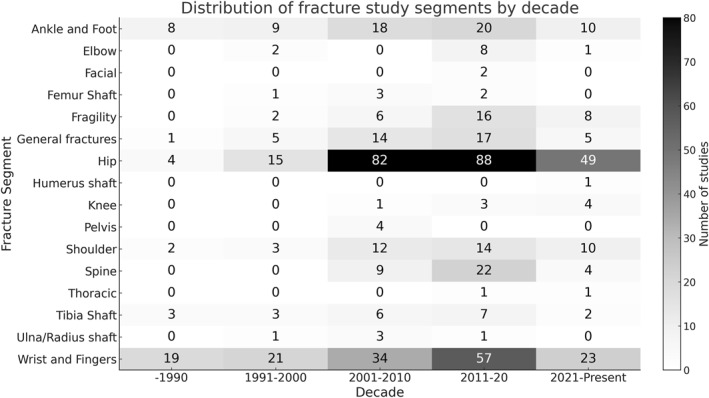
Heatmap illustrating the number of studies published per anatomical segment across different decades. Darker cells indicate a greater volume of research.

Analysis of study segments by decade revealed notable trends. Wrist and finger fractures remained the most frequently investigated across all periods, with a peak in 2011–2020 (*n* = 57). Hip fractures also showed substantial growth, particularly from 2001 to 2020, reflecting increased interest likely related to aging populations. Studies on fragility‐related and spinal fractures began to emerge after 2000 and grew steadily in subsequent decades. In contrast, other segments, such as the elbow, femoral shaft, thoracic, and ulna/radius shaft, were less frequently studied and showed no consistent pattern over time (Figure 1).

### 3.5 Clinical Trials Score and Geographic Distribution

**FIGURE 2:**
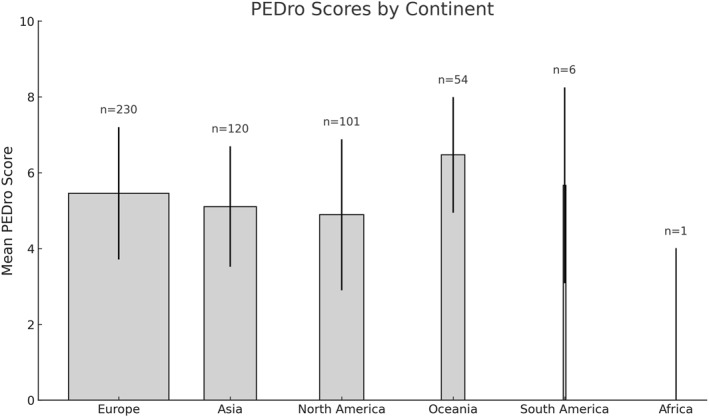
PEDro scores of clinical trials by continent, with bar widths proportional to the number of included studies. No data were available from Central America.

**FIGURE 3:**
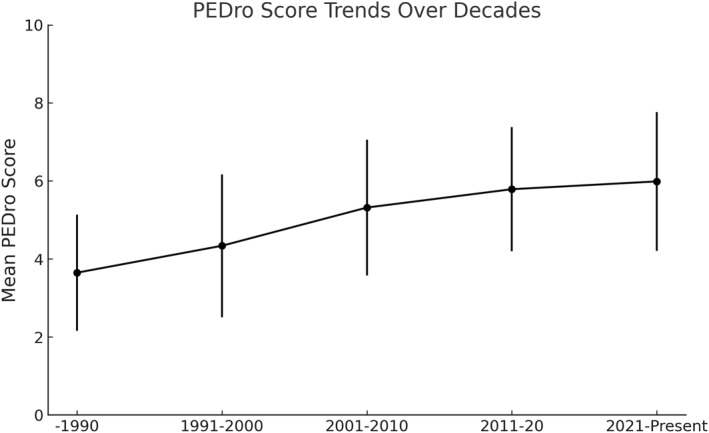
Temporal trends in PEDro scores of clinical trials.

Clinical trials (CTs) were conducted across all continents, with Europe hosting the largest number (*n* = 230), followed by Asia (*n* = 120), North America (*n* = 101), Oceania (*n* = 53), South America (*n* = 6), Africa (*n* = 1), and none from Central America. The continent of origin could not be identified for the two trials. Mean PEDro scores showed outstanding variation between regions. Oceania presented the highest average (6.47 ± 1.53), exceeding the scores observed in all other continents. South America also showed relatively high scores (5.67 ± 2.58) compared to the other continents, despite the small number of trials. Europe and Asia had similar averages (5.46 ± 1.74 and 5.11 ± 1.59, respectively), while North America recorded one of the lowest averages (4.89 ± 1.99). Africa had a score of 4 based on a single study (Figure 2). Over time, mean PEDro scores progressively increased from 3.65 ± 1.49 in studies published before 1990 to 4.34 ± 1.83 in 1991–2000, 5.32 ± 1.74 in 2001–2010, 5.79 ± 1.59 in 2011–2020, and 5.99 ± 1.78 in 2021–present (Figure 3).

PEDro scores also varied across anatomical segments. The highest mean score was observed in studies on thoracic fractures (7.00 ± 0.00), although this was based on only two trials and should be interpreted with caution. Among the more frequently studied segments, the highest averages were observed for shoulder (5.88 ± 1.96) and fragility‐related fractures (5.88 ± 1.54), followed by hip (5.66 ± 1.56) and spine (5.50 ± 1.61). Lower scores were found in studies on general fractures (4.35 ± 1.69) and ankle and foot (4.85 ± 2.11).

**FIGURE 4:**
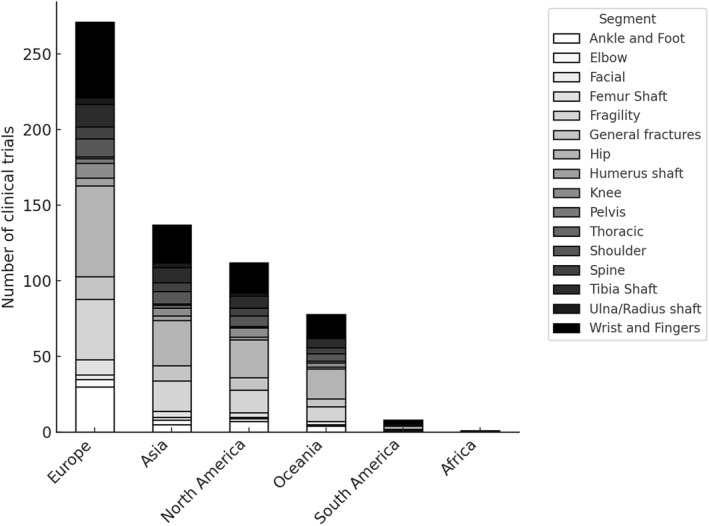
Distribution of anatomical fracture segments in clinical trials across continents. Stacked bars represent the number of clinical trials per segment in each continent.

When examining the anatomical focus of clinical trials across continents, distinct regional patterns emerged (Figure 4). In Europe and North America, most trials addressed hip fractures. Asia presented a more heterogeneous distribution, with notable representation of tibial shaft and ankle/foot fractures alongside hip fractures. Oceania demonstrated a more balanced profile across both upper and lower limb segments.

## 4 Discussion

This study aimed to map the characteristics of fracture rehabilitation studies indexed in the PEDro database, providing an overview of their distribution by anatomical segment, population, study design, continent, and decade of publication. Fractures represent a major cause of disability worldwide and are a common focus of physiotherapy practice, particularly in the context of population aging and increasing survival after trauma. However, fracture‐related studies accounted for only 3.2% of all PEDro records, highlighting a potential gap in the physiotherapy research landscape. Among the 667 included studies, lower limb fractures were the most frequently investigated (51.4%), followed by upper limb fractures (31.6%), with hip and wrist/finger fractures dominating their respective categories.

This relatively low representation of fracture rehabilitation research contrasts with the clinical relevance of fractures in physiotherapy practice. Although global fracture incidence has declined compared to previous decades, the absolute number of fractures has increased in recent years, mainly due to population aging, which increases the incidence of falls, and the growing burden of road traffic injuries (GBD 2019 Diseases and Injuries Collaborators 2020).

Only 12 clinical practice guidelines are indexed in PEDro, corresponding to hip, wrist, and fragility‐related fractures, indicating that several body segments lack this study design. Systematic reviews accounted for 141 studies covering more segments in their analysis. However, the CTs had an average PEDro score of 5.35, showing the low methodological quality of the studies dedicated to fractures according to the platform's classification. These results clarify the barriers clinical physiotherapists encounter in practicing the evidence‐based treatment of patients with fractures.

The results show that studies prioritize the hip and wrist/fingers segments. This can be explained by the high incidence of fractures and their clinical implications. The leading cause of these fractures is falls. In the case of hip fractures, the incidence is 681.35 (95% UI 508.36–892.27) per 100,000 inhabitants (Feng et al. 2024) in the population over 55, while distal radius fractures account for up to 17.5% of all fractures in adults (Court‐Brown and Caesar 2006). In addition, projections indicate a potential increase in various fractures related to osteoporosis and bone fragility due to population growth and aging (Borgström et al. 2020; Dong et al. 2022; Feng et al. 2024; GBD 2019 Fracture Collaborators et al. 2021). This priority is observed consistently across continents, although the relative proportion of other segments varies regionally, likely reflecting differences in local epidemiological patterns and health system priorities.

A different pattern is observed for other common fracture types, which remain relatively underrepresented in research output. Patella, tibia, fibula, or ankle were the most common fractures (GBD 2019 Fracture Collaborators et al. 2021), but they only accounted for 14,1% of fracture studies. In line with this reality, they also proved to be the biggest cause of years lived with disability, with 15.5 million in both genders (GBD 2019 Fracture Collaborators et al. 2021).

Our results indicate the most extensive scientific production related to the physiotherapeutic treatment of fractures in developed regions, such as Europe, Asia, North America, and Oceania. In contrast, scientific production could have been more limited in developing regions, including South America, Central America, and Africa. According to GBD 2019 Fracture Collaborators, the incidence of fractures in countries such as Brazil, Mexico, and most countries on the African continent is between 943.2 and 1347.5 per 100,000 inhabitants, while in developed countries such as Australasia region, United States, Canada, and much of the European continent, the incidence is between 2063.4 and 6407.3 per 100,000 inhabitants. However, many developing countries, including those in Central America, lack health metrics, and their health services do not cover most of the population, so the number of fractures may be underreported. Despite this, there is a tendency for the characteristics of fractures in these countries to be related to a higher level of energy and younger populations, such as in car accidents, accidents at work, or when playing sports, and also terrorism (GBD 2019 Fracture Collaborators et al. 2021). In addition to the imbalance in the volume of research, our findings also revealed disparities in methodological quality: Oceania achieved the highest mean PEDro score, while North America and Africa recorded the lowest averages. Overall, this pattern suggests a continuous rise in the scientific output on fracture management from the 1990s onward, with a possible plateau or stabilization in recent years.

Building research capacity and expanding the availability of robust evidence for rehabilitation are essential to support evidence‐based practice. Therefore, future research should focus on conducting clinical trials with greater methodological rigor for poorly studied fracture segments and issues, such as early mobilization and recovery of functionality, corroborating evidence‐based practice, and focusing on the patient and their contextual factors.

As a limitation, this study provides a descriptive analysis of the scientific literature on fracture care indexed in the Physiotherapy Evidence Database (PEDro), focusing on both thematic distribution and methodological quality as assessed by the PEDro score. By mapping the volume, type, anatomical focus, and quality ratings of the studies, we aimed to characterize the research profile available to physiotherapists through one of the most widely used evidence‐based platforms in the field. These findings should be interpreted as a reflection of the database's indexed content and not as a comprehensive synthesis of the entire literature on fracture rehabilitation.

## 5 Conclusion

Fracture studies represent a small portion of the PEDro platform. Most body segments lack clinical practice guidelines, and some do not have systematic reviews. Additionally, these studies have an average methodological quality below six points on the PEDro scale. The studies prioritize the hip and wrist segments, which are common fracture sites in the elderly. The peak in study production was observed in the 2010s. There is a geographic disparity in scientific production, led by continents with developed countries, especially Europe.

These findings underscore the significant barriers clinical physiotherapists face in applying EBP for patients after fractures. This call to action highlights the need for further research, guidelines, and support to improve patient outcomes and the quality of care in this area, particularly in segments with lower representation.

## 6 Implications for Physiotherapy Practice

These findings show that physiotherapists often face challenges in finding solid scientific support for fracture's rehabilitation management, particularly for certain anatomical regions. When evidence is scarce, practitioners must rely on their clinical reasoning and adapt interventions. Expanding the quality and scope of fracture research may offer clearer support for therapeutic decisions. As a result, physiotherapists in diverse settings would be better prepared to guide patients toward improved recovery and quality of life.

## Author Contributions

All authors contributed to the study conception and design. Ana Carolina Carmona Vendramim and Pedro Henrique Alberani Soares performed the data collection and conducted the statistical analysis. Aline Miranda Ferreira, Raquel M.M. Sugano and Marisa C.R. Fonseca interpreted the results. Ana Carolina Carmona Vendramim and Pedro Henrique Alberani Soares drafted the initial manuscript. Aline Miranda Ferreira, Raquel M.M. Sugano and Marisa C.R. Fonseca critically reviewed the manuscript for important intellectual content. All authors read and approved the final manuscript.

## Ethics Statement

The authors have nothing to report.

## Consent

The authors have nothing to report.

## Conflicts of Interest

The authors declare no conflicts of interest.

## Data Availability

The authors have nothing to report.
